# Enterovirus-Associated Hand-Foot and Mouth Disease and Neurological Complications in Japan and the Rest of the World

**DOI:** 10.3390/ijms20205201

**Published:** 2019-10-20

**Authors:** Gabriel Gonzalez, Michael J. Carr, Masaaki Kobayashi, Nozomu Hanaoka, Tsuguto Fujimoto

**Affiliations:** 1 Division of Bioinformatics, Research Center for Zoonosis Control, Hokkaido University, Sapporo 001-0020, Japan; gagonzalez@czc.hokudai.ac.jp; 2 National Advanced Computing Collaboratory, National Center for High Technology, San Jose 1174-1200, Costa Rica; 3 National Virus Reference Laboratory, School of Medicine, University College Dublin, D04 V1W8 Dublin, Ireland; michael.carr@ucd.ie; 4 Global Station for Zoonosis Control, Global Institution for Collaborative Research and Education (GI-CoRE), Hokkaido University, Sapporo 001-0020, Japan; 5 Kobayashi Pediatric Clinic, Fujieda 426-0067, Japan; koba-m@if-n.ne.jp; 6 Division 4, Infectious Disease Surveillance Center, National Institute of Infectious Diseases, 1-23-1 Toyama, Shinjuku-ku, Tokyo 162-8640, Japan; nozomu@nih.go.jp

**Keywords:** hand-foot and mouth disease, central nervous system complications, encephalitis, molecular characterization, enterovirus A71

## Abstract

Enteroviruses (EVs) are responsible for extremely large-scale, periodic epidemics in pediatric cohorts, particularly in East and Southeast Asia. Clinical presentation includes a diverse disease spectrum, including hand-foot and mouth disease (HFMD), aseptic meningitis, encephalitis, acute flaccid paralysis, and acute flaccid myelitis. HFMD is predominantly attributable to EV-A types, including the major pathogen EV-A71, and coxsackieviruses, particularly CV-A6, CV-A16, and CV-A10. There have been multiple EV-A71 outbreaks associated with a profound burden of neurological disease and fatal outcomes in Asia since the early 1980s. Efficacious vaccines against EV-A71 have been developed in China but widespread pediatric vaccination programs have not been introduced in other countries. Encephalitis, as a consequence of complications arising from HFMD infection, leads to damage to the thalamus and medulla oblongata. Studies in Vietnam suggest that myoclonus is a significant indicator of central nervous system (CNS) complications in EV-A71-associated HFMD cases. Rapid response in HFMD cases in children is imperative to prevent the progression to a CNS infection; however, prophylactic and therapeutic agents have not been well established internationally, therefore surveillance and functional studies including development of antivirals and multivalent vaccines is critically important to reduce disease burden in pediatric populations.

## 1. Introduction

Multiple, highly contagious Enterovirus A types are the etiological agents of infectious disease outbreaks affecting pediatric populations worldwide [1,2]. Among these types, enterovirus A71 (EV-A71), coxsackievirus A6 (CV-A6) and coxsackievirus A16 (CV-A16), are most frequently associated with hand-foot-and-mouth disease (HFMD), aseptic meningitis (AM) and encephalitis [2,3]. Despite the vast majority of enterovirus infections being sub-clinical and self-resolving in nature, large outbreaks caused by EV-A71, CV-A6, and CV-A16 have been reported in Asia since 1997 with severe cases developing complications affecting the central nervous system (CNS) with persisting sequelae and fatalities [4,5,6].

**Figure 1:**
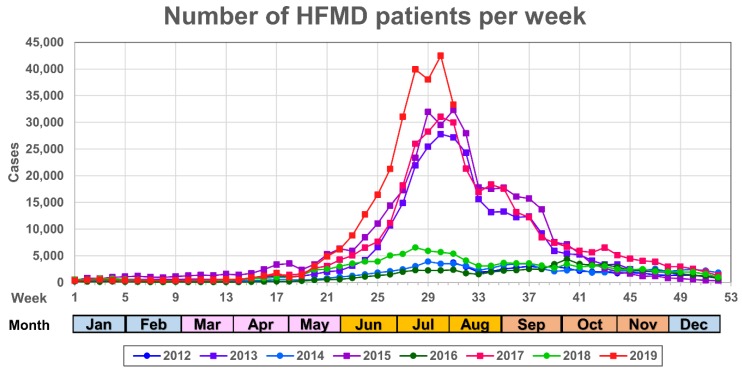
Distribution of the number of hand-foot and mouth disease (HFMD) cases per week in each year between July 2012 and August 2019 in Japan. The horizontal and vertical axes represent the week of the year and the number of cases in Japan for each week, respectively. Months corresponding to the week number are colored by Japanese season as spring (pink), summer (orange), autumn (light brown) and winter (light blue). Colors and marks for each yearly series are presented in the legend at the bottom of the panel.

**Figure 2:**
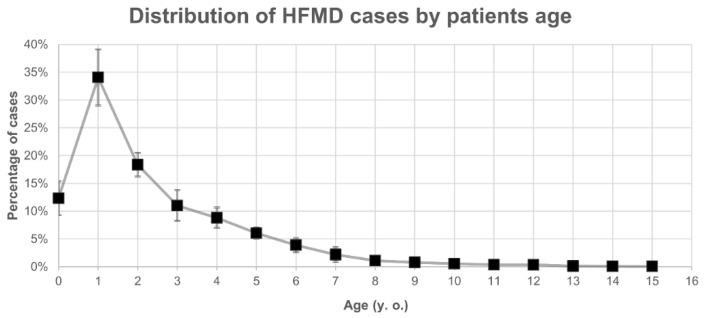
Distribution of HFMD cases by age between 2009 and 2018. The counts of patients were normalized as the percentages of the yearly total number of cases (vertical axis) and then stratified by age (horizontal axis).

Annual HFMD data are recorded by the National Epidemiological Surveillance of Infectious Diseases (NESID) in Japan [7] with 60% of the cases occurring between weeks 27 to 37 during the summer season (July and September in Figure 1). HFMD patients are typically children up to three years of age and represent 76% of the total number of annual cases (Figure 2). Among these cases, the severe infections comprised brainstem encephalitis and deaths from central pulmonary edema and heart failure [8]. As a consequence, HFMD outbreaks are of great public health importance as enteroviruses (EVs) can easily spread by droplet transmission and via fomites among children in day nurseries or kindergartens [9].

The present study details the current research related to the incidence, diagnosis, treatment and clinical management of HFMD, AM and encephalitis, arising from laboratory-confirmed enterovirus A types EV-A71, CV-A6, and CV-A16. Viral, epidemiological and clinical factors are analyzed to provide a comprehensive assessment of the pathology associated with EV infections and what parameters contribute to prevention and countermeasures to mitigate disease chains of transmission.

## 2. Enterovirus A

Species Enterovirus A are classified within the genus Enterovirus, one of the more than 30 genera in the family *Picornaviridae*, which also includes the related human viral pathogens within the Hepatovirus, Parechovirus, and Kobuvirus families [10]. Members of the *Picornaviridae* family have non-enveloped, 30–32 nm icosahedral capsids containing 60 tightly-packed protomers which are highly stable under acidic conditions allowing virions to survive exposure to gastric acid, disinfectants and remain infectious at ambient room temperature for several days [11]. The positive-sense, single-stranded RNA genomes, ranging from 6.7 to 10.1 kb in size, possess 5′ and 3′ untranslated regions (UTRs) and encode a single polyprotein from which all viral gene products are processed by viral proteases [10,12]. The proteins encoded in the enteroviral genome are organized from 5′ to 3′ with up to four structural capsid proteins followed by seven nonstructural proteins [10,12].

The Enterovirus genus consists of 12 species of Enterovirus A to J and Rhinovirus species A to C [10], among which Enterovirus A to D and Rhinovirus A to C have been isolated previously from humans. The genus contains strains from multiple taxa of polioviruses, coxsackieviruses (CVs) and echoviruses, based on biologic and antigenic characteristics during their original characterization [13]. Furthermore, despite polioviruses and CVs both producing diseases in the CNS, CVs were initially distinguished by their propensity to produce paralysis in suckling mice but no cytopathic effects in cultured cells [13]. Additionally, CVs were further divided into CV groups A and B according to the pathological characteristics observed in new-born mice, with members in CV-A causing flaccid paralysis and CV-B causing spastic paralysis [14]; however, polioviruses and echoviruses produce cytopathic effects in cultured cells while failing to cause lesions in new-born mice [13]. However, problems arose from this biological classification scheme as different strains could exhibit properties of different taxa; therefore, from EV-D68, and subsequent newly recognized EVs, are named by assigning a chronological number in order of discovery [10,13]. EVs have traditionally been serotyped by assessment of neutralizing antibody responses in cultured cells; however, since 2000, DNA sequence-based genotyping approaches have become standard practice, with a “type” rather than a serotype name, e.g., Echovirus type 30 rather than Echovirus serotype 30. In recent years, the genetic typing of specimens has grown due to the more rapid and simpler PCR testing for EV detection [15,16], while use of cultured cells are also increasingly being supplanted by molecular approaches due to their low isolation rates. For the identification of the viral type by nucleotide sequence analysis, the VP4-VP2 partial region of the capsid region and the analysis by amplifying the VP1 partial region (CODEHOP RT-semi-nested PCR) are widely used. In general, EV type identification by sequencing of the VP1 region, which is highly concordant with serotype designations, is considered reasonable [17]. Currently, more than 300 EV types have been characterized genetically by phylogenetic clustering, including rhinoviruses [10,18]; in detail, Enterovirus A: 25 types, Enterovirus B: 63 types, Enterovirus C: 23 types, Enterovirus D: 5 types, Enterovirus E: 5 types, Enterovirus F: 7 types, Enterovirus G: 20 types, Enterovirus H: 1 type, Enterovirus I: 1 type, Enterovirus J: 6 types, Rhinovirus A: 80 types, Rhinovirus B: 32 types, Rhinovirus C: 55 types, and three types awaiting formal classification [10,18]. Pathogens associated with HFMD outbreaks belong to the species Human enterovirus A (HEV-A). In addition, in the context of CNS infections, poliovirus causing poliomyelitis (acute flaccid paralysis, AFP), which remarkably has almost achieved vaccinal control and eradication worldwide [19], is classified as Enterovirus C. EV-D68, which has caused large-scale respiratory infections in Japan, Europe and North America since 2014, and has been definitively associated with multiple paralytic cases (acute flaccid myelitis) arising from spinal cord damage, is classified within species Enterovirus D [20,21,22,23].

The species HEV-A consists of CV A2-A8, A10, A12, A14 and A16, and EV-A71 [14]. The EV-A71 prototype (Br-CA-70 strain) was first described in 1969 in the USA from cases presenting with neurological disease [24,25]. Most of the HFMD cases worldwide are caused by HEV-A viruses and severe cases develop complications involving the CNS and respiratory tract [26,27]. HFMD is in most cases a self-limiting illness, however, a small proportion of cases (<1%) develop life-threatening neurological and systemic complications [19]. Additionally, other infections by HEV-A members are herpangina, AM, meningoencephalitis, acute flaccid paralysis, gastroenteritis, and encephalitis [19,27].

## 3. Enterovirus Infection

EVs are predominantly transmitted through the fecal-oral route, i.e., susceptible individuals contact with fecal material from infected individuals that gains access to the oral mucosal epithelium. Additionally, EVs are also transmitted through inhalation of saliva and respiratory droplets in sneeze, cough, drip or other respiratory exhalations from infected individuals. It is noteworthy that expelled droplets can spread in a 1 m^2^ range dependent upon ambient humidity [28,29]. Additionally, other fluids from infected individuals, such as secretions from blisters in HFMD cases, can also readily transmit the virus directly or via fomites [30].

After an EV gains entry to the oral epithelia by contact with contaminated material, the virus infects the oropharynx and subsequently the gastrointestinal tract. The infection can proliferate in the tonsils, cervical lymph nodes, Peyer’s patches, and the mesenteric lymph nodes [29,31]. The incubation period is usually 3 to 6 days, although some are as short as 1 to 3 days, such as in acute hemorrhagic conjunctivitis cases due to CV-A24 [29]. Viral shedding from the respiratory tract is usually within 7 days [32]. Dependent upon the EV type and severity of the infection, shedding of the virus in feces may persist for > 10 days [2]. In our experience, it is extremely rare that the virus is isolated from feces more than two weeks after disease onset. There are also EV types that caused acute respiratory infections in Japan in 2015 that are difficult to isolate from feces, such as EV-D68, which is associated with an acute flaccid paralysis/myelitis presentation, in rare but serious cases, and for which droplet infection is the main route of infection and therefore a respiratory specimen is recommended for laboratory diagnosis [33]. As the clinical signs start to manifest in different organs, viremia becomes detectable and the risk of sepsis increases [31]. In severe cases, as the viremia increases, the virus spreads to skin, mucous membranes, respiratory tract, heart, liver, pancreas, and, in some cases, enters the CNS and neurological and cardio-respiratory failure can potentially develop [8,30,31].

## 4. Hand, Foot, and Mouth Disease (HFMD)

**Figure 3:**
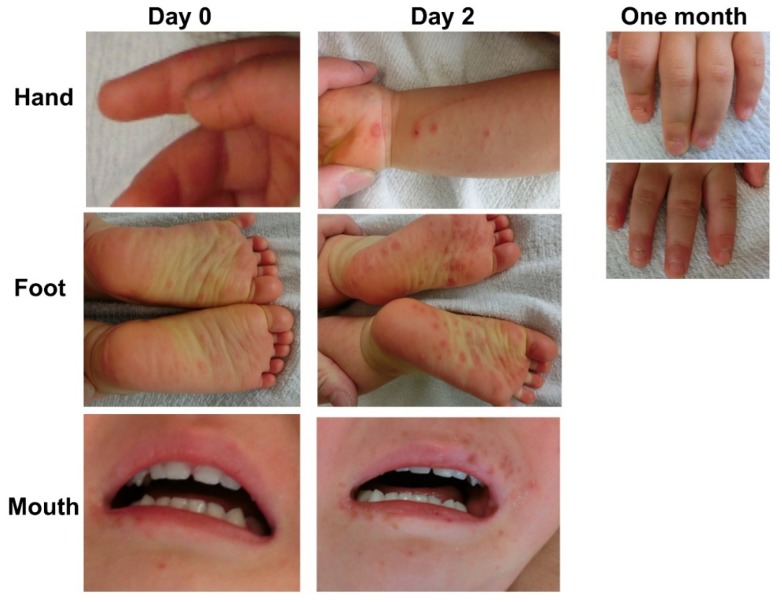
Photographs of a patient with the HFMD caused by CV-A6. Day 0 and 2 after the onset of the disease in hands, feet, and mouth. After one month, onycholysis was observed for both hands (foot and mouth lesions healed after one month). Pictures were taken with the informed consent of patients.

HFMD was first discovered in North America as an acute-onset, febrile viral infection with a characteristic maculopapular rash and blisters observed on the hands, feet, and mouth [31]. Inflammation and vesicles in the mouth can ulcerate with associated pain and discomfort that complicates eating and drinking for infants which can lead to dehydration. While most cases are self-limiting, a small percentage can develop further neurological complications [11]. Other symptoms include nausea, vomiting, sore throat, fatigue, malaise, loss of appetite, irritability, upper respiratory tract infection, gastroenteritis and non-specific viral rashes [19,26,31]. Despite the highly infectious nature of HFMD pathogens, which results in the high number of infections per outbreak, the symptoms usually resolve 7 to 10 days after disease onset [34]. HFMD cases are most prevalent in the summer (Figure 1) and the main etiological agents are members of the species Enterovirus A: CV-A16, and EV-A71, with some less frequent cases caused by CV-A6 and CV-A10 [2]. Since 2009, atypical HFMD cases caused by CV-A6 have been reported in Japan every other year from 2011 to 2019. The trend of cases caused by CV-A6 has been attributed to an inferred five-fold increase in pathogenicity since 2010; such an increase provided a better fit to the data than models involving changes in transmissibility or antigenicity [35]. Moreover, these cases are considered atypical due to symptoms that could be misdiagnosed as varicella, such as vesiculobullous rash of the trunk and extremities, and the characteristic spread of eruptions with scabbing and crust formation that can leave skin lesions [27,36] (Figure 3).

## 5. EV Infections in the Central Nervous System

EV infection can produce a high titer viremia that extends the infection from the primary site in the enteric or respiratory tissue to the CNS resulting in diverse pathologies. Among such pathologies, poliovirus acute poliomyelitis caused by members of the species Enterovirus C has been considered among the most serious epidemic diseases. Poliomyelitis complications include spinal or bulbar paralysis with consequent respiratory failure [27]. Nevertheless, as of 2019, poliovirus is close to global eradication with some exceptions in Pakistan and Afghanistan [27,37]. Less than 1% of individuals infected with EVs are infected with types belonging to Enterovirus C and cause CNS disease; on the other hand, about 4 to 8% of infected individuals recover with only a febrile illness alone, and more than 90% are considered asymptomatic [38]. A HFMD survey of 7.2 million Chinese cases from 2008 to 2012 reported a fatality rate of 0.03% (2000) of cases and severe complications in 1.1% (>70,000) of cases [39].

As vaccination efforts have almost eradicated poliovirus, EV-A71 has become the most frequently associated EV to cause severe CNS infections among the non-polioviruses and has emerged as the “new polio” in Asia [2,19,40]. AM and encephalitis are among the most common manifestations of CNS infections by EVs. In the period between 2011 and 2012, encephalitis or encephalopathy was reported in 23 out of 2471 cases (0.93%) by EV-A71 in the Infectious Disease Survey in Japan. On the other hand, encephalitis or encephalopathy was reported in 10 out of 3202 patients (0.31%) infected with CV-A16 during the same period. Although virus detection in patients with encephalitis does not definitively indicate the causative infectious agent, this figure suggests that CV-A16 may also cause encephalitis and encephalopathy and warrants further investigation. 

Human fatalities and mice experiments involving EV-A71 infections in the CNS suggest the virus traverses the blood-brain barrier via retrograde axonal transport along cranial or peripheral nerves [41]. CNS involvement in HFMD cases is typically seen 1–5 days after infection; early symptoms include headache, lethargy, suckling weakness, irritability, limb tremors, myoclonic twitches, vomiting, and myopathy. Despite this, most cases will resolve without consequence. However, in a proportion of clinical cases, after prolonged illness (>5 days) with increased blood pressure, elevated heart and respiratory rates, severe cases can develop cardiopulmonary failure. Symptoms in this stage include tachycardia, tachypnoea, cyanosis, cough with bloody sputum and hypotension. The progression of these symptoms leads to high mortality without timely diagnosis and treatment that is also only effective during the early stages of infection therefore early diagnosis and treatment is critically important [30].

CNS inflammation by EV-A71 is found in the grey matter of the spinal cord, throughout the medulla oblongata, the dorsal nucleus and ciliary body of the vagus nerve, hypothalamus, subthalamic nucleus, and dentate nucleus. Although less severe than these, inflammation is also seen in the motor cortex of the cerebrum. On the other hand, there are no inflammatory changes in the cerebellar cortex, thalamus, basal ganglia, peripheral nerves, and autonomic ganglia. In addition, histopathological changes, similar to effects caused by other viruses, include perivascular cellular invasion, edema, neuronophagia, and microglial nodules. However, the presence of viral particles is not observed, though viral antigens and nucleic acids have been identified in a small number of neurites and phagocytes [11]. Fulminant forms of pulmonary edema are thought to be preceded by CNS symptoms and lead to death from EV-A71. It remains unclear whether increased vascular permeability or cytokine storm influences the outcome. The concomitant occurrence of encephalitis and HFMD is difficult to predict; moreover, encephalitis is often unclear and prospective studies are difficult.

EV infections are also reported to cause paralysis along with encephalitis [17]. Before May 2018, Japanese surveillance lacked diagnosis and surveillance for AFP cases; therefore, during the AFP outbreak of 2015, an additional epidemiological survey based on notification from the Ministry of Health, Labour and Welfare was conducted [33]. Although EV-D68 has been suspected as a significant contributor of AFP cases [42], the association with paralysis makes EV-A71 the current major AFP pathogen [43].

## 6. Surveillance of HFMD and AM in Japan

HFMD and AM across Japan are part of the diseases under surveillance in the NESID (available at https://www.niid.go.jp/niid/en/iasr-e.html), according to the Japanese Infectious Diseases Control Law, with more than 3000 fixed points in pediatric clinics and hospitals nationwide with weekly reports (Figure 1). Clinical samples are collected in ~10% of pediatric sites, and viruses are detected and identified by molecular methods in local health laboratories nationwide [8]. As the main causative agents of HFMD, EV-A71 and CV-A16 have been monitored across Japan between 2012 and 2018 (Table 1); it is noteworthy that every three years a rise in the number of cases has been noted and attributed to a three year cyclical component of EV-A71 [44], however, closer inspection to the distribution of etiologic agents also suggests that CV-A16 cases rise and fall periodically. Furthermore, EV-A71 was the main cause of epidemic outbreaks in 2000 and 2003 in Japan [2], while CV-A16 was reported as the cause of outbreaks in Japan and also in India [45]. On the other hand, a variety of EVs were detected as a cause of AM in Japan between 2012 and 2018, among them types in Enterovirus A. It is noteworthy that the annual number of detected AM cases is on average 0.8% ± 0.6 of the total number of HFMD cases, which is remarkable considering the biannual rise in the number of cases in the latter. The number of recorded AM events, similar to the number of HFMD cases, peaks in summer. However, the causative EV pathogens associated with AM belong on average to Enterovirus B in 74% ± 15 of cases per year, with the mumps paramyxovirus (which is not part of the routine Japanese vaccination) the second most common cause in 16% ± 9 of cases (Table 1). Therefore, factors common to Enterovirus A and B and the number of susceptible/previously exposed individuals in the population determine the number of cases, together with other factors, such as the average temperature and other prevailing weather conditions promoting the spread of these viruses in the summer months [46]. Nevertheless, EV-A71 was the etiologic agent in ~10% of AM cases in 2013 and 2017 coinciding with two peaks of infection reflecting the risks associated with outbreaks from this neurotropic EV. EV-induced meningitis has a fundamentally favorable prognosis (particularly in comparison to bacterial meningitis) so timely laboratory confirmation of aseptic meningitis is crucial for clinical care.

Although HFMD is reported in 90% of cases in infants under 5 years of age (Figure 1), AM outbreaks are more frequently reported in patients in the age range 5 to 15 years [27]. However, due to the high number of patients with EV infections under 15 years and the percentage of cases developing AM, EV surveillance has been focused on pediatric populations and the prevalence of infections in adults remains underreported; it is noteworthy that atypical HFMD by CV-A6 infections has been also reported in adults [47,48].

EV-induced HFMD is recognized clinically as a syndrome; however, EV-induced CNS infections, such as encephalitis, are more frequently judged as encephalitis of unknown origin unless the pathogen is confirmed by laboratory methods. HFMD was identified as a distinct clinical entity 60 years ago, and since then has been a significant source of outbreaks [24,49,50,51,52,53,54,55,56,57,58,59,60,61,62,63,64,65]. During this time, numerous mutations in the EV genome have arisen resulting in a diversity of genotypes with consequent changes in antigenicity and pathogenicity, as shown in animal models [66]. Phenotypic variation, such as differences in antigenicity between strains and the possibility of recombination among EV types, suggests the emergence of new types is ongoing [67,68].

## 7. Enterovirus Detection and Isolation

Although rapid point-of-care diagnostic kits are available and frequently used in Japan in clinical practice for other viruses, such as adenovirus in ocular infections [69], no such kits have become currently available for EVs [70]. In the clinical context, other viral tests besides rapid diagnosis, are rarely performed for viral infections in Japan. Recently, PCR devices are available at Japanese hospitals, and rapid screening systems became available in 2018 [71]. Pathogen testing is mandatory to clarify the cause of the disease in cases showing complications; moreover, for the appropriate implementation of pathogen testing the proper selection, collection, storage, and transport of clinical specimens are critically important. 

Usually, EV-A71 is difficult to detect from cerebrospinal fluid (CSF), and PCR tests are often possible from feces and throat swabs. Fecal and throat samples can also be frozen and stored until molecular testing [15]. Despite the neurological complications associated with EV-A71, the detection rate from CSF is low in EV-A71-associated meningitis cases. Nevertheless, simultaneous viral testing in the acute phase from throat swabs and feces is useful for the identification of causative pathogens [32]. In the case of post-mortem studies, the lesion inferred from the imaging diagnosis is collected from the peripheral tissue responsible for the brain function. After harvest, it is rapidly divided into nucleic acid extraction (frozen), virus isolation culture (frozen at −80 °C) and histopathological examination (10% neutral buffered formalin-fixed at room temperature) for storage. In cases of EV-caused encephalitis, sampling of the brainstem regions, such as the thalamus and medulla oblongata, is mandatory.

## 8. Molecular Characterization of EV-A71

**Figure 4:**
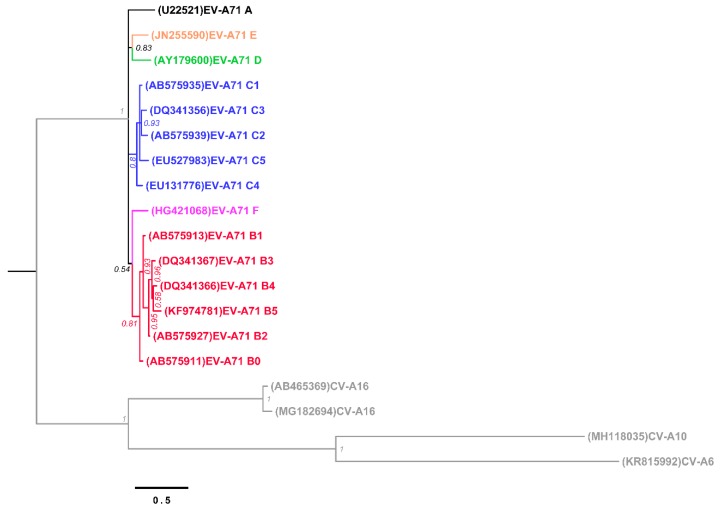
Phylogenetic tree of nucleotide sequences encoding the VP1 in EV-A71 genogroups, CV-A6, CV-A10, and CV-A16. The phylogenetic tree was inferred with MrBayes v3.5 [78] with the general time reversible substitution model allowing for heterogeneity modeled by a gamma distribution and allowing for invariable sites (GTR + G + I) with 5 × 10^6^ chain length. The sequences are publicly available in GenBank with the accession number between parentheses. Tips are colored according to the genogroup A, B, C, D, E, F, and CV genotypes (CV-A6, -A10 and -A16), as black, red, blue, green, orange, purple and gray, respectively. Posterior probability supporting the topology is shown next to the branches.

Strains of EV-A71 are frequently characterized into genogroups based on the sequence of the VP1, which represents the major epitope determinant and binding properties of the viral particles [72]. Currently, there are six genogroups from A to F [73], and genogroups B and C are further subdivided into B0 to B5 and C1 to C5, respectively (Figure 4). The genogroup A contains the prototype EV-A71, and genogroups D, E and F contain a single strain each. The high strain diversity in EV-A71 is attributed to a high evolutionary rate, ca. 4 × 10^−3^ substitutions per site per year estimated on the VP1 [74]; such a high mutation rate is due to the lack of a proofread mechanism in the viral RNA-dependent RNA polymerase (RdRp) with overall mutation rates of 10^−3^ to 10^−5^ mutations per copied nucleotide per replication cycle [75]. Additionally, despite recombination events in the capsid-encoding region are considered infrequent due to structural and functional constraints, the effects of recombination in EV-A71 are considered source of diversity among strains and genogroups [73,76,77]. The VP1 divergence and the evolutionary forces modeling a growing diversity of EV-A71 are important factors to consider in the development and deployment of vaccines [75] It is noteworthy that the capsid region exhibits low recombination rates, while the 5′UTR-VP4 junction and P2 have been shown to represent recombination boundary hotspots [77].

## 9. Treatment and Vaccines against HFMD

Asian countries with endemic EV infections and histories of large epidemics are actively developing vaccines as prophylactics. As a national project in China, a vaccine against EV-A71 was developed and introduced to the market in 2016 [30,79]; furthermore, in a 12-month analysis of EV-A71 infections in healthy children between 6 and 35 months, infections in 0.3% (13/5041) of the vaccinated group compared with 2.1% (106/5028) of the placebo group were described. 

On the other hand, drugs targeting EV-A71 have been developed, but therapeutics principally employs immunological approaches with high-dose gamma-globulin which has limitations in its practical use: (1) the use of IVIG has yet to be supported by evidence from randomized clinical trials, (2) IVIG is not without risk, due to the use of human blood products and the significant infusion volume required, and (3) it is prohibitively expensive [2,80]. In principle, treatments for EV infections are palliative and focused on combating the symptoms alone. The use of glucocorticoids should be considered with prudence as reports suggest limiting their usage to cases in which antiviral therapy fail to control the clinical picture [81,82]. Despite a lack of specific drugs for clinical treatment of HFMD, small molecules have been used to inhibit EV-A71 infections by blocking the polymerase such as ribavirin and DTriP-22 [83,84].

## 10. Risk Factors for Severity in HFMD Cases

Among the HFMD etiologic agents, EV-A71 is the most pathogenic and more frequently associated with complications extending to the CNS, such as brainstem encephalitis [85,86]. Moreover, severe cases with EV-A71 infections could be detected earlier if caution is exercised in patients with physical findings, raised total white cell count, vomiting and absence of mouth ulcers [87]. Although fever is common to EV-A71 infections, its duration and severity have been shown as important independent risk factors [2]. Other early signs related to CNS complications are lethargy, agitation or irritability, myoclonic jerk, truncal ataxia and rotary eye movement without fixation [2,88]. According to a meta-analysis, duration of fever ≥ 3 days, body temperature ≥ 37.5 °C, lethargy, hyperglycemia, vomiting, increased neutrophil count, EV-A71 infection, and young age are risk factors for severe HFMD [89]. Although formalin-inactivated vaccines against EV-A71 have been developed in several Asian countries, an EV-A71 vaccine does not provide protection against other heterologous types, such as CV-A16, CV-A6, and CV-A10 [31,90]. Mono- and bivalent vaccines have been developed to CVs and shown to be efficacious [91,92,93] and quadrivalent vaccines including EV-A71, CV-A16, CV-A6, and CV-A10 which afford balanced immunity and shown to be safe and effective in humans after clinical trials would dramatically reduce the HFMD and neurological disease burden and the associated socioeconomic costs. 

A multivariate analysis of the severity of HFMD considering 459 severe and 246 mild cases of HFMD, respectively, in China, found four risk factors more frequently in severe cases: (1) fatigue (*p* < 0.01, OR = 204.7); (2) the use of glucocorticoids (*p* = 0.03, OR = 10.44); (3) the use of dehydrating agents (*p* < 0.01, OR = 73.7), and (4) a maculopapular rash (*p* < 0.01, OR = 84.4) [91]. Other reports also suggest the use of steroids in acute encephalitis cases associated with EV-A71 clearly exacerbate central nervous system damage [82].

## 11. Summary

This review outlines the burden imposed by EV-associated HFMD and the development of CNS complications, such as encephalitis and meningitis. HFMD is often caused by EV-A71, CV-A6, and CV-A16 and there have been multiple extremely large outbreaks with associated neurological disease and fatal outcomes due to EV-A71 infections in East and Southeast Asia since the early 1980s. Vaccines against EV-A71 have been developed and used in China, which has suffered the highest burden of disease and fatalities, but widespread pediatric vaccination programs have not been introduced in other countries. Although fatal cases have been reported in Japan, the absolute number of severe cases caused by EV-A71 remains lower than those in China, Malaysia, Singapore, and other Asian countries. Nevertheless, baseline epidemiological surveillance in Japan of this infectious disease provides insights into the infectious trends of EV-A71 to assist in the breaking of chains of transmission and mitigating disease events.

Neurological complications arising from a HFMD infection lead to brainstem encephalitis and lesions in the thalamus, medulla oblongata and other CNS tissues. Studies in Vietnam suggest that myoclonus is a significant indicator of early neurological disease in EV-A71-associated HFMD cases.

Rapid response against HFMD in children is imperative to prevent the progress of the infection into the CNS, as the latter has serious outcomes. As prophylactic and therapeutic agents have not been well established internationally, surveillance, and functional studies including development of new antivirals and balanced multivalent vaccines is critically important to reduce disease burden in pediatric populations. 

## Figures and Tables

**Table 1:** HFMD and aseptic meningitis (AM) cases detected per year and the causative agents.

Disease	Virus *	2012	2013	2014	2015	2016	2017	2018
HFMD		46530	301071	83219	379368	68834	355686	122041
	EV-A71	46%	82%	43%	0%	22%	64%	65%
	CV-A16	54%	18%	57%	100%	78%	36%	35%
AM		453	1058	854	1035	1332	928	760
	*Enterovirus A*	15%	11%	0%	17%	0%	19%	12%
	CV-4	1%	-	-	-	-	-	-
	CV-6	-	2%	-	-	-	5%	-
	CV-9	12%	-	-	17%	-	4%	4%
	EV-A71	2%	9%	-	-	-	10%	7%
	*Enterovirus B*	66%	81%	92%	71%	91%	52%	65%
	CV-B1	-	3%	-	-	3%	-	-
	CV-B2	-	-	7%	3%	2%	10%	3%
	CV-B3	-	13%	5%	4%	8%	-	-
	CV-B4	2%	2%	4%	-	-	2%	12%
	CV-B5	13%	9%	12%	9%	41%	-	9%
	Echovirus 11	-	-	29%	-	-	-	25%
	Echovirus 16	-	-	-	4%	-	-	-
	Echovirus 18	1%	6%	9%	33%	4%	-	8%
	Echovirus 3	-	-	7%	3%	2%	6%	-
	Echovirus 30	-	22%	15%	5%	6%	-	-
	Echovirus 6	25%	26%	3%	-	20%	20%	4%
	Echovirus 7	14%	-	-	-	-	3%	3%
	Echovirus 9	11%	-	-	10%	4%	10%	-
	Mumps virus ^+^	19%	7%	8%	12%	9%	30%	23%

* Percentages of cases per type are calculated based on sampled cases by the NESID. ^+^ The paramyxovirus mumps is also a causative agent of aseptic meningitis in Japan and is included in this table for comparison with the number of cases attributable to EV types.

## Abbreviations

EV	Enterovirus
CV	coxsackievirus
HFMD	hand-foot and mouth disease
AM	aseptic meningitis
CNS	Central Nervous System
NESID	National Epidemiological Surveillance of Infectious Diseases
HEV-A	*Human enterovirus A*
AFP	acute flaccid paralysis
CSF	cerebrospinal fluid
RdRp	RNA-dependent RNA polymerase
